# ATM is activated in blood monocytes after tumor radiation

**DOI:** 10.18632/oncotarget.1286

**Published:** 2013-08-14

**Authors:** Andrei V. Gudkov, Elena A. Komarova

**Affiliations:** Department of Cell Stress Biology, Roswell Park Cancer Institute, Buffalo, NY USA; Department of Cell Stress Biology, Roswell Park Cancer Institute, Buffalo, NY USA

Almost one million of cancer patients receive radiotherapy in USA every year. Therefore, it is very important to estimate their therapeutic responses to radiation and potential injury of normal tissues which can be manifested later in life [1]. Measure of potential radiation damage is also critically important in the event of a nuclear accident or terrorism efforts. Conventional approaches which allow to measure radiation damage and based on the evaluation of radiation-induced changes in blood chemistry and cell count and cytogenetic assessment of chromosomal aberrations [2] do not satisfy to contemporary demands of radiation medicine. Therefore, developing of new approaches evaluating radiation damage is acutely important in our days. Earlier we found that radiation induced expression of specific set of genes that encoded secreted bioactive proteins and were p53-dependent [3]. Moreover, we detected radiation-induced changes in activities of a number of transcription factors (TF) in mouse liver using transfection *in vivo* of TF plasmids developed by Attagene Inc. (Gudkov, Komarova, Makarov, personal observations). Acute response proteins and inflammatory cytokines, such as C-reactive protein, IL-6 [4] were detected in blood of animals after radiation. It was found that several proteins change their expression or undergo post-translational modifications after radiation and can be considered as putative markers of radiation exposure including CDKN1A (cyclin-dependent kinase inhibitor 1A), GADD45A (growth arrest and DNA-damage-inducible 45 alpha), BLM (Bloom syndrome protein), Tp53 (tumor protein p53), H2AX (Histone 2AX) and ATM (ataxia telangiectasia mutated) [5]. ATM, The Ser/Thr protein kinase, is known for its role as a main mobilizer of the cellular response to a radiation-induced severe DNA lesions, double-strand breaks (DSBs). In undamaged cells, quiescent ATM exists as homodimers, which dissociate into active monomers upon activation [6]. It was shown that autophosphorylation at Ser 1981, post transcriptional modification of ATM, is a hallmark of activated human ATM. It is still unclear what is the initial trigger of ATM activation. It was suggested that a chromatin conformational change that follows DSB formation rather than direct contact of ATM with broken DNA might activate ATM. Another studies suggested that direct interaction of ATM with broken DNA is required for its activation [6].

In the recent paper, Bakkenist et al [7] have found that ATM kinase activity is induced in monocytes of peripheral blood of cancer patients after first high dose fraction of Stereotactic body radiation therapy (SBRT), which was delivered to specific tumor targets including non-small cell lung cancer, pancreatic adenocarcinoma, renal cell carcinoma and gastroesophageal adenocarcinoma. This is the first demonstration of ATM serine-1981 phosphorylation and activation of ATM in patients following radiation. The authors detected activation of histone H2AX but in less degree in monocytes of the same patients. A similar picture they observed *in vitro* titrating dose-dependent induction of ATM and H2AX in cancer cell lines. It would be expected that ATM might be activated as a result of chemotherapeutic treatment in blood monocytes as well (the authors did not test this) because it was shown that ATM might be activated not only by DSBs but by another genotoxic stresses (for example single strand brakes) and nongenotoxic stresses including hypoxia, hyperthermia, oxidative stress [6]. Moreover, it was shown that oxidative stress induced by phorbol myristate acetate was associated with intense phosphorylation of histone H2AX and with ATM activation in human peripheral blood leukocytes [8].

The data presented here show that ATM activation may be an excellent biomarker for exposure to radiation (and other agents) in human patients and may be used in predicting of their therapeutic response. Also, it is clear that even targeted local radiation therapy induces systemic DNA damage response seen in particular as activation of ATM. Systemic inhibition of ATM as the important member of DNA repair complex might increase the efficacy of targeted radiotherapy that is confirmed by recently developed ATM inhibitors (KU-55933, CGK733, and CP466722) that increased radiosensitization of tumor cells [9].
